# Gum Lancing

**Published:** 1893-05

**Authors:** 


### Gum-Lancing.

Clinically, I am absolutely sure that I have seen convulsions, sick stomach, great restlessness, fever, and various other functional disturbances in young children, immediately cured by the use of the gum lancet, after the failure of various other well directed measures for relief. Theoretically, I am in accord with Dr. Kirk in believing that Dr. Forchheimer absolutely misses the point of the matter, by his failure to understand that the good achieved is not due to the local blood-letting or to the relief of the inflammation of the gum, but to the removal of the backward pressure upon an extraordinarily sensitive and (at such times) congested tooth-pulp. As was long ago pointed out by Dr. J. W. White, at the period of eruption the roots of the teeth are incomplete.

I have myself seen a seemingly incurable epilepsy in an adult permanently cured by the removal of a persistent milk or first dentition tooth. Amaurosis and various other conditions in the adult are well known to be the result of irritation of the trigeminal nerve by faulty teeth. How much more is evil to be expected from teeth-irritation in the child?

In conclusion, I reaffirm that whatever the theory in the matter may be, I am positive that gum-lancing is the most important therapeutic measure. It is essential, however, that it should be thorough, and with the object of dividing the dense tissues that bind down the teeth.—*University Medical Magazine.*

